# Acute hospital staff’s attitudes towards dementia and perceived dementia knowledge: a cross-sectional survey in Ireland

**DOI:** 10.1186/s12877-020-01783-6

**Published:** 2020-09-30

**Authors:** Brian Keogh, Wing Ting To, Louise Daly, Geralyn Hynes, Siobhan Kennelly, Brian Lawlor, Suzanne Timmons, Susan O’Reilly, Mairead Bracken-Scally, Aurelia Ciblis, Natalie Cole, Amanda Drury, Chiara Pittalis, Brendan Kennelly, Mary McCarron, Anne-Marie Brady

**Affiliations:** 1 School of Nursing and Midwifery, The University of Dublin, Trinity College, 24 D’Olier Street, Dublin, D02 Ireland; 2 Medicine for the Elderly, Connolly Hospital, Blanchardstown and National Clinical Integrated Care Programme, Health Services Executive, Dublin, Ireland; 3 Director Mercer’s Memory Clinic, St James’s Hospital, Dublin 8, School of Medicine and Global Brain Institute, Trinity College Dublin, Dublin, Ireland; 4 Centre for Gerontology and Rehabilitation, School of Medicine, University College Cork and Mercy University Hospital, Cork, Ireland; 5 Medicine for the Elderly, Connolly Hospital, Blanchardstown, Dublin, Ireland; 6 National Dementia Office, Health Service Executive, Dublin, Ireland; 7 National Research and Development Office, Health Service Executive, Dublin, Ireland; 8 Department of Epidemiology and Public Health Medicine, Royal College of Surgeons in Ireland, Dublin, Ireland; 9 School of Business and Economics, National University of Ireland Galway, Galway, Ireland

**Keywords:** Dementia, Staff attitudes, Acute hospital, Dementia knowledge, Approaches to dementia questionnaire

## Abstract

**Background:**

Little is known about staff’s attitudes in Irish acute hospital settings towards people living with dementia and their perceived dementia knowledge. The aim of this study was to understand the general level of dementia knowledge and attitudes towards dementia in different types of hospital staff, as well as to explore the potential influence of previous dementia training and experience (having a family member with dementia) and the potential moderating effects of personal characteristics. This data was required to plan and deliver general and targeted educational interventions to raise awareness of dementia throughout the acute services.

**Methods:**

A cross-sectional survey was carried out among a diverse range of hospital staff (*n* = 1795) in three urban acute general hospitals in Ireland, including doctors, nurses, healthcare attendants, allied professionals, and general support staff. Participants’ perceived dementia knowledge and attitudes were assessed as well as their previous dementia training and experience. To measure participant’s attitude towards dementia, the validated Approaches to Dementia Questionnaire (ADQ) was used.

**Results:**

Hospital staff demonstrated positive attitudes towards people living with dementia, and believed they had a fair to moderate understanding of dementia. Both ‘having previous dementia training’ and ‘having a relative living with dementia’ predicted attitude towards dementia and perceived dementia knowledge. Interestingly, certain personal staff characteristics did impact dementia training in predicting attitude towards dementia and perceived dementia knowledge.

**Conclusion:**

This study provides a baseline of data regarding the attitudes towards dementia and perceived dementia knowledge for hospital staff in Irish acute hospitals. The results can inform educational initiatives that target different hospital staff, in order to increase awareness and knowledge to improve quality of dementia care in Irish hospitals.

## Background

Dementia is a leading cause of disability and dependency among older people [1]. It is estimated that there are approximately 50 million people living with dementia worldwide [2]. In Ireland, there are approximately 55,000 people estimated to live with dementia [3, 4]. This figure is expected to increase to 151,157 persons by 2046 [5] and represents a significant social and economic challenge to society, policy and service delivery [6]. It is estimated that about one-third of older people in acute Irish hospitals may have dementia [7–9]. However, the acute hospital setting can often be experienced as disorientating and stressful by people living with dementia and previous research has indicated that this can result in adverse health and functional outcomes including the provision of insufficient care [9–19]. Furthermore, limited knowledge and understanding of dementia among general hospital staff, coupled with organisational constraints on a busy hospital ward and traditional task approaches to care within acute settings, can contribute to negative attitudes towards people living with dementia and challenges to the ability to provide person-centred care [11, 13, 14, 20, 21]. This creates a culture of care where patient safety and risk are prioritized over patient dignity, care is disease/illness orientated and people living with dementia are seen as a low priority [13, 14]. In addition, there is a lack of understanding and knowledge of person-centred care within the acute hospital setting [13] resulting in poorer experiences and outcomes for people living with dementia [9–19].

Previous studies have shown that, despite availability of a national education programme specifically for acute hospital staff, the provision and uptake of such education in Irish acute hospitals is poor [15, 22] and that staffing levels and lack of resources may be a barrier to staff attending dementia training [15]. The Irish National Dementia Strategy [9] has proposed some actions to address the needs of people living with dementia in a more responsive and individualised manner. One of the actions proposed is to build more dementia awareness and understanding.

Studies have demonstrated that staff who have a good knowledge of dementia care have more positive attitudes towards people living with dementia [23, 24], which in turn have been suggested to be associated with better quality of care [24–29]. Thus, it has been proposed that high quality of care for people living with dementia is dependent on staff having a high level of dementia knowledge and positive attitudes towards people living with dementia [24] (e.g. being more optimistic about people living with dementia and perceiving those people as sentient human beings). It has also been shown that dementia training programs can improve staff knowledge, attitudes and confidence in caring for people living with dementia [24, 30–34]. Travers and colleagues (2013), for instance, showed that previous training in dementia is a predictive factor for more positive attitudes towards people living with dementia, which they have assessed using the validated questionnaire ‘Approaches to Dementia Questionnaire’ (ADQ) [35]. Furthermore, Kada and colleagues (2009), using the same measure, demonstrated that staff that had previous specialised training in dementia had significantly higher ‘hope’ attitudes compared to staff who had not undertaken such training [36]. Staff with more hopeful attitudes are more likely to engage in activities and social interactions with people living with dementia that are based around the principles of person-centred care [35].

Besides dementia training, studies have investigated whether exposure to dementia in one’s work or family (personal experience of dementia) predicted more positive attitudes towards dementia and increased dementia knowledge. Regarding dementia knowledge, the study by Carpenter and colleagues (2011) demonstrated that exposure to dementia through one’s work or family is related to enhanced dementia knowledge [37]. However, the findings with regards to the influence on attitudes towards dementia are unclear. The study by Cheston and colleagues (2016) suggested that individuals with personal experience of dementia held more positive attitudes towards dementia than those with no experience of dementia [38], whereas McParland and colleagues (2012) demonstrated that the experience of knowing someone with dementia did improve dementia knowledge [39], but contrary to what one might expect, it was not a strong indicator for a more positive attitude. A recent study using the validated measure Approaches to Dementia Questionnaire (ADQ) to assess participants’ attitudes towards dementia demonstrated that increased contact with people living with dementia was associated with increased ADQ scores reflecting more hope and person-centred attitudes towards dementia [40].

Research has focused on attitudes of professionals specifically working with people living with dementia [35, 41, 42] as well as on the attitudes of the general public [38, 39]. However, to our knowledge, there is little known about the attitudes of general hospital staff towards people living with dementia, their perceived knowledge, experience and education in acute general hospitals in the Republic of Ireland. This information will firstly provide an indication of hospital staff readiness to support people living with dementia and their understanding of personhood and person-centred care [4, 9]. In addition, understanding knowledge of and attitudes to dementia will assist in the design of educational initiatives to build dementia awareness and to provide a baseline to measure other planned improvements in dementia friendliness across hospital settings. The aim of this study was to examine attitudes to dementia and their perceived knowledge of dementia in all types of hospital staff in three large acute hospitals (clinical as well as non-clinical staff) in Ireland.

## Methods

The study provides baseline data regarding attitudes toward dementia and their perceived dementia knowledge for hospital staff, against which changes over time can be assessed and compared to. Additional aims of the study reported herein, were to investigate whether having previous training in dementia or having personal experience with dementia (i.e. having a relative with dementia) is associated with differences in attitudes towards dementia or in their perceived knowledge regarding dementia. Furthermore, demographic factors were investigated to examine the impact they might have, through dementia training and/or personal experiences with dementia, in predicting their attitude towards people living with dementia (ADQ score) or their perceived dementia knowledge.

### Design, setting and participants

This study utilized a cross-sectional survey design where data was collected in three urban acute general hospitals in Ireland from August 2014 through September 2015. These hospitals were undergoing a range of service developments to improve the provision of care to people living with dementia. The data reported herein were collected as part of one aspect of the overall evaluation of these initiatives. The survey was available to complete electronically through a survey link of SurveyMonkey [43] that was sent to staff email addresses via a gatekeeper. Hard copy surveys were also available in all patient care areas to complete and return to a central location via internal mail for those without access to or unable to use a computer [40, 44]. All grades of staff including support staff (e.g. administrative, catering, domestic and security staff) were invited to take part in the survey as they might have been involved in the care of people living with dementia. The study was advertised via staff email, posters, newsletters and announcements at staff meetings to maximise awareness of the study so as to enhance the response rate/participation.

### Survey

The survey consisted of the Approaches to Dementia Questionnaire (ADQ) [34] and a dementia knowledge question as our two outcome measures.

#### ADQ

The Approaches to Dementia Questionnaire is a validated questionnaire that aims to assess participants’ attitudes towards dementia [35], and has been shown to be reliable, easy to administer and to score [40–42, 45]. The ADQ is a 19-item survey that assesses attitudes towards people living with dementia using a five-point Likert scale ranging from ‘strongly agree’ to ‘strongly disagree’. The total ADQ score ranges from 19 to 95, with higher scores reflecting more positive attitudes towards people living with dementia. Factor analyses have shown that the ADQ comprises of two domains or attitudes: *hope* and *person-centred* attitudes [35]. The *hope* subscale consists of 8 items reflecting a sense of optimism/pessimism about the abilities and the future of the people living with dementia. The *person-centred* subscale consists of 11 items reflecting the extent to which people have a person-centred understanding of dementia or recognize and respond to people living with dementia as unique individuals with the same value as any other person. The ADQ score in our study showed good internal consistency with the overall value of the Cronbach’s α being .78. For the subscale *hope* the Cronbach’s α was .70 and for the *person-centred* attitude .77.

#### Dementia knowledge

To assess participants’ perceived knowledge of dementia, they were asked to tick their overall knowledge of dementia on a 10-point scale ranging from 1 - ‘I know nothing at all’ to 10 - ‘I am very knowledgeable’. It was assumed that the participants perceived knowledge of dementia may influence their attitudes towards dementia.

In addition, two other dementia specific questions were asked to assess potential predictors for ADQ and dementia knowledge, namely whether participants had any previous training in dementia (‘Have you had previous training/education in dementia?’) and whether they had been personally affected by dementia in their environment (‘Do you have, or have you had, a family member with dementia/Alzheimer’s Disease?’). Finally, in terms of demographics, participants were asked to report their gender, age group and job role.

### Ethical considerations

Ethical approval was granted by the Research Ethics Committees of Trinity College Dublin Faculty of Health Sciences Research Ethics Committee. Ethical approval was also received from the Health Service Providers where the research was conducted. Participants received a participant information leaflet and were informed that a return of a completed survey implied consent to participate in the study. Researchers were bound by and adhered to national and international codes of ethical practice including rules regarding informed consent, data management and storage.

### Data analysis

Data from the online questionnaire was downloaded from SurveyMonkey [43] into Excel and data collected on paper were manually entered into Excel. Data from both sources were then transferred to SPSS 25 [46] for all statistical analysis.

Cronbach’s α was used to assess the internal consistency of the total and subscale ADQ measures. Descriptive statistics were computed for demographic characteristics and for the ADQ and dementia knowledge summary scores. Multiple regression analyses were carried out to identify whether ‘previous dementia training’ and ‘having a relative with dementia’ are important predictors for their attitude towards people living with dementia as well as for perceived dementia knowledge. In order to investigate potential impact that demographic factors might have on the participant’s dementia training and personal experiences with dementia in predicting their attitude towards people living with dementia (ADQ score) or their perceived dementia knowledge, a series of two-way Univariate analysis of variances (ANOVAs) were conducted to investigate potential interaction effects with gender (male versus female), age (18–54.9 years old versus 55–75 years old) and job roles (doctors, nurses and healthcare attendants versus allied professionals versus general support staff). Allied professionals included allied health professionals (non-specified), physiotherapists, dieticians, social workers, pharmacists, speech and language therapists, radiographers, occupational therapists, physiologists, psychologists, and orthopaedic technicians. General support staff included administrative staff, laboratory, management, chaplain, hospital catering, hospital housekeeping, security, maintenance, shop, porter amongst others.

## Results

### Participant’s characteristics

A total of 1795 hospital staff completed the survey. However, the sample size is reported in parenthesis if there was missing data. It was not possible to determine the response rate as the survey was distributed widely and the total number of survey recipients is unknown. The demographic characteristics of the participants are displayed in Table 1. Participants were mostly female (83.2%, *n* = 1791) and aged between 35 and 57 years (56.7%, *n* = 1246). Approximately a quarter of the participants did not provide their age range. The most common discipline of those who answered the question (*n* = 1783) was nursing (41.4%). We merged the occupations into meaningful groups for analysis, these being ‘doctors, nurses and healthcare attendants’ (50.8%, *n* = 906), ‘allied professionals’ (11.8%, *n* = 210) and ‘general support staff’ (37.4%, *n* = 667). Most staff reported not having any previous dementia training (76.5%, *n* = 1791) and more than half (62.1%, *n* = 1474) indicated that they do not have a relative with dementia.

**Table 1:** Characteristics of hospital staff

Variable	n (%)
Age	N = 1246
- 18–35 years	421 (33.79%)
- 35–55 years	706 (56.66%)
- 55–75 years	119 (9.55%)
Gender	N = 1791
- Male	300 (16.8%)
- Female	1491 (83.2%)
Job Role	N = 1783
- Doctors, nurses, healthcare attendants	906 (50.8%)
- Allied professionals	210 (11.8%)
- General support staff	667 (37.4%)
Previous dementia training	N = 1791
- Yes	420 (23.5%)
- No	1371 (76.5%)
Family member with dementia	N = 1474
- Yes	558 (37.9%)
- No	916 (62.1%)

### Attitudes toward dementia

#### Descriptives

The average total ADQ score was 70.64 (*Sd* = 8.60) (maximum total ADQ score = 95), with an average item score of 3.72 (i.e. the total score divided by the number of items) (maximum average item score = 5). The average total *hope* subscale score was 24.88 (*Sd =* 5.41) (maximum total hope subscale score = 40) with an average item score of 3.11 (maximum average item score = 5) and the average total *person-centred* subscale score was 45.77 (*Sd* = 5.16) (maximum total person-centred subscale score = 55) with an average item score of 4.16 (maximum average item score = 5). A paired sample t-test on the average subscale scores revealed a significantly higher average score for the ADQ subscale *person-centred* attitude compared to *hope* attitude (*t*(1776) = − 142.66, *p* < .001).

#### Multiple regression

The multiple regression analysis indicated that both ‘previous dementia training’ (*β* = 4.61, *SE* = .51, *t* = 9.10, *p* < .001) and ‘having a relative with dementia’ (*β* = 2.50, *SE* = .46, *t* = 5.40, *p* < .001) both significantly contributed to a positive attitude towards dementia as measured with the ADQ (R^2^ = .07, *F*(2,1458) = 56.46, *p* < .001).

#### Moderator effects

A series of two-way ANOVAs were conducted to investigate whether ‘previous dementia training’ or ‘having a relative with dementia’ had an impact on the participants’ attitude towards dementia (*hope* subscale score, *person-centred* subscale score and total ADQ score) within different demographical groups, namely gender (male versus female), age (18–54.9 years old versus 55–75 years old) and job roles (doctors, nurses and healthcare attendants versus allied professionals versus general support staff).

**Fig. 1:**
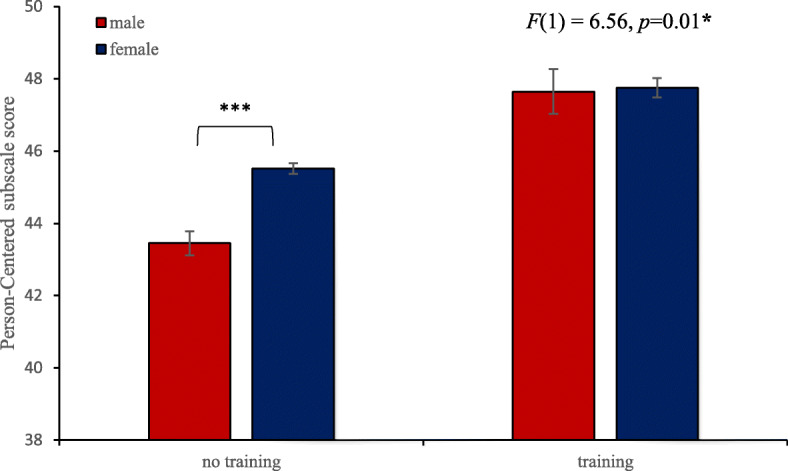
Interaction between ‘previous dementia training’ and gender for the Person-Centred subscale

The two-way ANOVAs examining whether the interaction between ‘previous dementia training’ and gender had an impact on attitude towards dementia did not result in a significant interaction effect for the *hope* subscale score (*F*(1,1770) = 0.58, *p* = .45), but did show a significant interaction effect for the *person-centred* subscale score (*F*(1, 1772) = 6.56, *p* = .01) (see Fig. 1). For the interaction between ‘dementia training’ and gender, contrast analysis revealed that males who did not have previous dementia training scored significantly lower on the *person-centred* subscale (*M* = 43.45, *Sd* = 5.67) compared to females who did not have previous dementia training (*M* = 45.52, *Sd* = 4.8) (*F*(1, 1772) = 32.48, *p* < .001). However, for participants who had previous dementia training, there was no difference in *person-centred* subscale scores found between males and females based on contrast analysis (*F*(1, 1772) = .02, *p* = .88). For the interaction ‘previous dementia training’ and gender on the total ADQ score the effect was close to significance (*F*(1, 1766) = 3.97, *p* = .05).

**Fig. 2:**
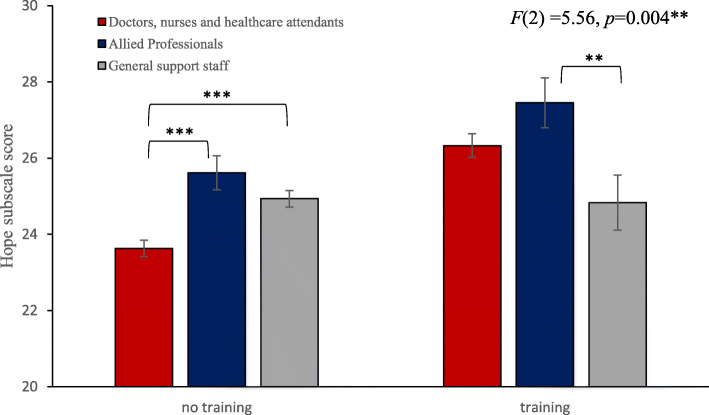
Interaction between ‘previous dementia training’ and job role for the Hope subscale

Regarding the interaction ‘previous dementia training’ and ‘job role’, a significant interaction effect was demonstrated for the *hope* subscale (*F*(2,1761) = 5.56, *p* = .004) (see Fig. 2). For the interaction of ‘previous dementia training’ and job role for the *hope* attitude, contrast analysis showed that there was a significant difference in hope subscale scores between having had previous training versus having no previous training for the doctors, nurses and healthcare attendants (*F*(1,1761) = 51.26, *p <* .001) and for the allied professionals (*F*(1,1761) = 5.37, *p* = .002), where the hope subscale scores were higher for the group that had previous dementia training. For staff who had previous dementia training, contrast analysis only showed differences between allied professional and general support staff (*F*(1,1761) = 7.22, *p* = .007), where allied professionals scored significantly higher on the *hope* subscale score (*M* = 27.45, *Sd* = 5.56) compared to the general support staff (*M* = 24.83, *Sd* = 6.19). No differences were found in the *hope* subscale score between the other job roles for staff who had previous dementia training using contrast analysis: doctors, nurses and healthcare attendants versus allied professionals (*F*(1,1761) = 4.42, *p* = .12) and comparing doctors, nurses and healthcare attendants versus general support staff (*F*(1,1761) = 3.62, *p* = .06). For staff who did not have previous dementia training, differences in *hope* subscale score were demonstrated using contrast analysis when comparing doctors, nurses and healthcare attendants with allied professionals (*F*(1,1761) = 15.95, *p* < .001) and when comparing doctors, nurses and healthcare attendants with general support staff (*F*(1,1761) = 18.11, *p* < .001), where doctors, nurses and healthcare attendants (*M* = 23.63, *Sd* = 5.86) scored significantly lower compared to the other job roles (allied professionals (*M* = 25.62, *Sd* = 5.52) and general support staff (*M* = 24.93, *Sd* = 4.36)), while no differences in hope subscale scores were found between allied professionals and general support staff (*F*(1,1761) = 1.91, *p* = .17).

### Perceived dementia knowledge

#### Descriptive results

The average score on the perceived dementia knowledge questions was 4.63 (*Sd* = 1.82) on a total scale of 10.

#### Multiple regression

Results of the multiple regression analysis indicated that both ‘previous dementia training’ (*β* = 1.49, *SE* = .10, *t* = 14.87, *p* < .001) and ‘having a relative with dementia’ (*β* = .36, *SE* = .09, *t* = 3.92, *p* < .001) both significantly contributed to their perceived dementia knowledge (R^2^ = .14, *F*(2, 1455) =118.71, *p* < 0.001).

#### Moderator effects

A series of two-way ANOVAs were conducted to investigate whether ‘previous dementia training’ or ‘having a relative with dementia’ have an impact on their perceived dementia knowledge within different demographic groups, namely gender (male versus female), age (18–54.9 years old versus 55–75 years old) and job roles (doctors, nurses and healthcare attendants versus allied professionals versus general support staff).

**Fig. 3:**
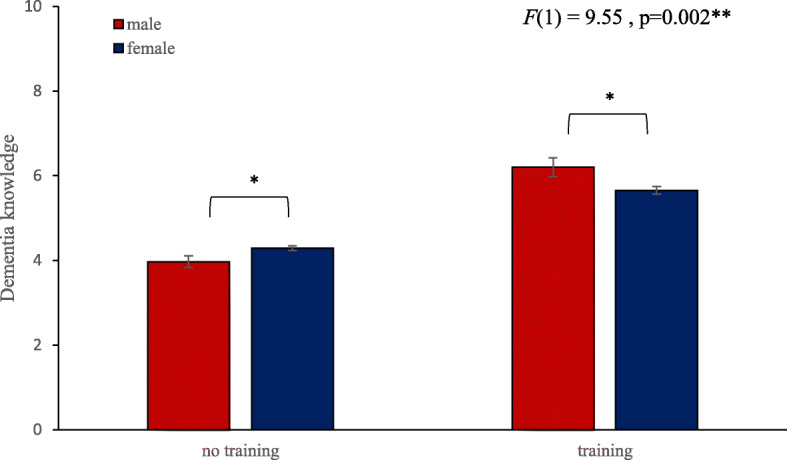
Interaction between ‘previous dementia training’ and gender for dementia knowledge

The two-way ANOVA examining whether the interaction between ‘previous dementia training’ and gender had an impact on dementia knowledge resulted in a significant effect (*F*(1,1455) = 9.54, *p* = .002) (Fig. 3). Contrast analysis demonstrated that for staff who did not have previous training males had a significantly lower perceived dementia knowledge score (*M* = 3.97, *Sd* = 1.63) compared to females (*M* = 4.29, *Sd* = 1.65) (*F*(1,1455) = 4.68, *p* = .03). For staff who had previous dementia training females had a significantly lower perceived dementia knowledge score (*M* = 5.66, *Sd* = 1.79) compared to men (*M* = 6.2, *Sd* = 1.80) (*F*(1,1455) = 5.26, *p* = .02).

Regarding the interaction between ‘previous dementia training’ and age (*F*(1,1322) = .002, *p* = .96) and the interaction between ‘previous dementia training’ and job role (*F*(2,1457) = .13, *p* = .88), no interaction effects were demonstrated on perceived dementia knowledge. Furthermore, investigating the interactions between ‘having a relative with dementia’ and gender (*F*(1,1454) = 1.60, *p* = .21), between ‘having a relative with dementia’ and age (*F*(1,1321) = .27, *p* = .60), and between ‘having a relative with dementia’ and job role (*F*(2,1456) = .34, *p* = .72), did not result in any significant interaction effects on perceived dementia knowledge.

## Discussion

The aim of this study was to explore the attitudes towards dementia as well as the perceived dementia knowledge of hospital staff in three large, urban Irish acute general hospitals. We investigated whether having previous training in dementia or having personal experience with dementia would predict participant attitudes towards dementia or their perceived dementia knowledge and whether demographic factors would impact on participants’ dementia training and personal experiences with dementia in predicting their attitude towards people living with dementia (ADQ score) or their perceived dementia knowledge.

Overall, the study demonstrated a largely positive attitude of hospital staff towards people living with dementia and a fair to moderate understanding of dementia based on participants’ own judgment. Both ‘having previous dementia training’ and ‘having a relative living with dementia’ predicted attitude towards dementia and perceived dementia knowledge. Interestingly, certain personal staff characteristics such as gender and job role did impact dementia training in predicting attitude towards dementia and perceived dementia knowledge.

Our findings in hospital staff indicated a largely positive attitude towards people living with dementia with a mean ADQ score of 70.64, comparable to the research findings in similar settings (acute hospital, mean ADQ score of 71.44, [47]; acute medical and orthopaedic wards, mean ADQ score of 72.7, [48] and different settings (nursing homes and hospital geriatric wards, mean ADQ score of 70.4, [36] . More specifically, the general positive attitude found in our study had a higher emphasis on person-centred attitudes than on the hope attitude which is in line with previous findings [24, 25, 36, 38, 45, 49, 50]. Even though our findings, similar to previous studies, do demonstrate positive attitudes to dementia among all hospital staff, the actual experience of the people living with dementia and their family can differ. Studies have shown that families commonly report negative care experiences in acute care environments [9, 21, 51, 52]. A diagnosis of dementia has been shown to be associated with stigma and as a result people living with dementia are reported to be at risk of sub-standard health care with evidence of protracted admission and discharge periods in acute care settings [53]. Such a diagnosis is also associated with higher prevalence of co-morbidities and those living with dementia with co-existing conditions are less likely to receive parity of care compared to people without dementia [54]. Bail (2013) demonstrated that Australians with dementia admitted to acute hospitals for either surgical or medical related problems develop higher rates of preventable complications such as urinary tract infections, pressure areas, pneumonia and delirium [55]. Dewing, & Dijk (2016), observed the tension between prioritisation of acute care within hospitals over the individual needs of the person living with dementia [13]. Evans (2018) attributes such inequalities to hospital culture that is focused on the challenges of dementia associated care rather than the personalised needs of the individual and this manifests in the attitudes of staff [53]. With regards to the participants’ perceived knowledge of dementia, hospital staff in our study reported only fair to moderate understanding of dementia, similar to previous findings [56].

Our findings support the hypothesis that previous dementia training and having personal experience with dementia (having a family member with dementia) predict a more positive attitude towards dementia. In addition, dementia training and having personal experience also predicted higher perceived dementia knowledge. As this study has indicated that both dementia education and having personal experience with dementia contributes to a more positive attitude towards dementia, educational initiatives could be set up that engage staff on an emotional level to optimize their capacity to deliver person-centred care to people living with dementia as suggested by Cowdell and colleagues (2010) [57]. It is suggested that effective education of healthcare staff is critical to the provision of high-quality care for people living with dementia, but there are considerable logistical challenges for acute hospital settings. A range of educational interventions have been found to be effective in influencing the behaviour of staff around person-centred care strategies in dementia [30, 32], but they have been designed for small numbers of staff. Hospitals are challenged by the need to train large numbers and diverse staff who are in contact with people living with dementia.

When exploring the interaction of personal characteristics of staff and previous dementia training in predicting their attitudes towards dementia and their perceived dementia knowledge, the study demonstrated a few interesting interaction effects. Firstly, gender interacted with ‘previous training’ in shaping attitudes towards people living with dementia and also on perceived dementia knowledge. For staff who did not have previous dementia training, females showed a more person-centred attitude compared to males. However, for staff who had previous dementia training, there was no difference in person-centred attitudes between males and females. This finding suggests that dementia training may moderate to neutralize gender differences in the person-centred understanding of dementia. A previous study by MacDonald and Woods (2005) suggested that increased person-centred attitudes are associated with better recognition of cognitive impairment in nurses, that is independent of training and experience [49]. However, this study supports the importance of training in increasing person-centred attitudes, which in turn could be associated with better recognition of cognitive impairment in people living with dementia. For their perceived dementia knowledge, similarly, males reported significantly lower scores than females, within the cohort of staff who did not have previous dementia training. In contrast, in staff who had previous dementia training, female staff reported lower scores on the perceived dementia knowledge question than males. Keeping in mind that dementia knowledge was not assessed using an objective dementia knowledge questionnaire, the perceived dementia knowledge question used in this study might have been influenced by their level of self-confidence or their awareness of their own level of dementia knowledge. For example, female staff could feel less confident and more aware of their poor dementia knowledge after dementia training, whereas male staff would feel more confident. In addition, the findings could be reflective of the high number of females in direct caregiving roles who may be more attuned to the realities of caring for people living with dementia within the acute services. In 2014, a study in Irish acute hospitals indicated that nurses at ward level are aware of their poor dementia knowledge and are open to dementia training [22]. Future studies might consider this gender difference as it could influence their openness to dementia training.

Secondly, our data showed significant higher hope scores for all staff who had previous dementia training compared to all staff who did not have previous training, except for the general support staff, where prior training did not seem to have an effect. This finding suggests that general dementia training might not influence the hope attitude of general support staff in hospitals towards people living with dementia, possibly reflecting the content of such education and training. Future dementia training might benefit from specifically focusing on increasing the hope attitude in this subgroup of hospital staff. For staff without any previous dementia training, the group of doctors, nurses and healthcare attendants were more pessimistic about dementia compared to the group of allied professionals and the group of general support staff, with notably increased hope in doctors, nurses and healthcare attendants with prior training. This group might benefit from dementia training focusing on influencing their hope attitude. For staff who had previous dementia training, allied professionals were significantly more hopeful then general support staff.

### Strengths and limitations

To our knowledge, this is the first large-scale study conducted in Irish acute general hospitals empirically assessing all staff attitudes towards dementia and dementia knowledge. The study is significant as it comprised all types of job roles in acute general hospitals, including clinical as well as non-clinical staff such as domestic staff and administrators. The majority of studies to date have focused on clinical staff. Including all hospital staff is important as a broad range of staff interact with people living with dementia, not only staff working intensively with the target population. Conceptualizing dementia care beyond health and social care speaks to the need to consider a whole systems and community approach when designing services for people living with dementia [9]. It should be noted that all types of staff (clinical or non-clinical) will have different education and knowledge needs which need to be considered when planning dementia awareness initiatives. In addition, specific pedagogical approaches will need to be considered in line with staff need and individual hospital requirements. Despite the strength of this study some potential limitations must be acknowledged. Firstly, given the high number of female respondents across the three hospitals involved, care must be taken when interpreting the findings of this study especially where the role of gender is considered. Secondly, our definition of previous experience with dementia was set to having a family member living with dementia. This could have been broadened by adding a friend or acquaintance with dementia [39]. We could also have added more detail to specify the extent of contact with the person with dementia, such as asking whether they had cared for the person with dementia [58], as a caring role could potentially influence attitudes in a complex way. Thirdly, a subjective measure of assessing dementia knowledge was used which rated dementia knowledge from one to ten. Adding an objective measure of dementia knowledge would have given us more objective findings [24]. Furthermore, a validated dementia knowledge scale would provide more understanding where specific gaps in dementia knowledge exist (e.g. disease prevalence, symptoms, risk factors, prevention, assessment or treatment) to more specifically guide educational initiatives to help sensitize individuals to what they do not know about dementia [38]. Thirdly, the study might have benefitted from having more specific information or factors explaining the level of dementia awareness and knowledge in hospital staff, including overall educational level [36, 41], whether they worked or were in contact with people living with dementia on a daily basis [40] or their confidence level working with people living with dementia [31].

## Conclusions

This study provides an understanding of the general level of dementia knowledge and attitudes towards dementia in different hospital staff in Irish acute hospital settings. In addition, the study explored if having previous dementia training or having a relative with dementia impacted on attitudes and/or knowledge. The findings reveal that both *having previous dementia training* and *having a relative living with dementia* predicted positive attitudes towards dementia and higher perceived dementia knowledge. However, staff discipline and gender moderate these effects. This study provides useful data to inform educational initiatives to increase awareness and knowledge and improve the quality of dementia care in Irish hospitals. It also underscores the importance of training and education to promote positive attitudes towards dementia.

## Data Availability

The dataset will not be made available; while the data is anonymized, the dataset contains a number of variables relating to hospital and clinicians and patient characteristics, which taken together, potentially increase the risk of identifying the individuals/hospitals involved.

## Abbreviation

ADQ: Approaches to Dementia Questionnaire
